# Will online behavioral research follow the fate of online survey research?

**DOI:** 10.1073/pnas.2535585123

**Published:** 2026-02-18

**Authors:** S. Van der Stigchel, C. Strauch, B. de Zwart, L. Van Maanen

**Affiliations:** ^a^Helmholtz Institute, Department of Experimental Psychology, Utrecht University, Utrecht 3584CS, The Netherlands

Westwood (1) convincingly shows that large language model-based agents can pass as human participants in online surveys, calling into question the reliability of self-report research. However, the threat may be considerably more pervasive than suggested. Using data from an online Posner cueing task, we find patterns consistent with AI-generated responding in reaction time (RT) datasets: a domain long thought to be safeguarded by perceptual-motor constraints. If bots can now mimic not only opinions but also millisecond-level behavior, the foundations of online experiments in cognitive science are equally at risk.

In a recent online Posner cueing experiment (39 participants on Prolific), we identified several participants whose response profiles were highly consistent with simulated, bot-generated data rather than human performance (details on the experiment and analysis can be found on Open Science Framework: https://osf.io/7mz9x). Using parameters derived from the real dataset (mean RT, SD), we simulated RT-distributions under simple Gaussian assumptions (200 trials/condition; no intertrial dependency). We then compared each participant to these simulations across three markers:

1. Distributional shape (Q–Q deviation): Suspected bots showed near-perfect normality; humans showed the expected rightward skew (2).
2. Mean-SD scaling: Typical human responders showed a positive mean-variance relationship; suspected bots showed no such trend (3).
3. Autocorrelation: Human RTs normally exhibit serial dependence; suspected bots showed near-zero autocorrelation across trials (4).

**Fig. 1.**
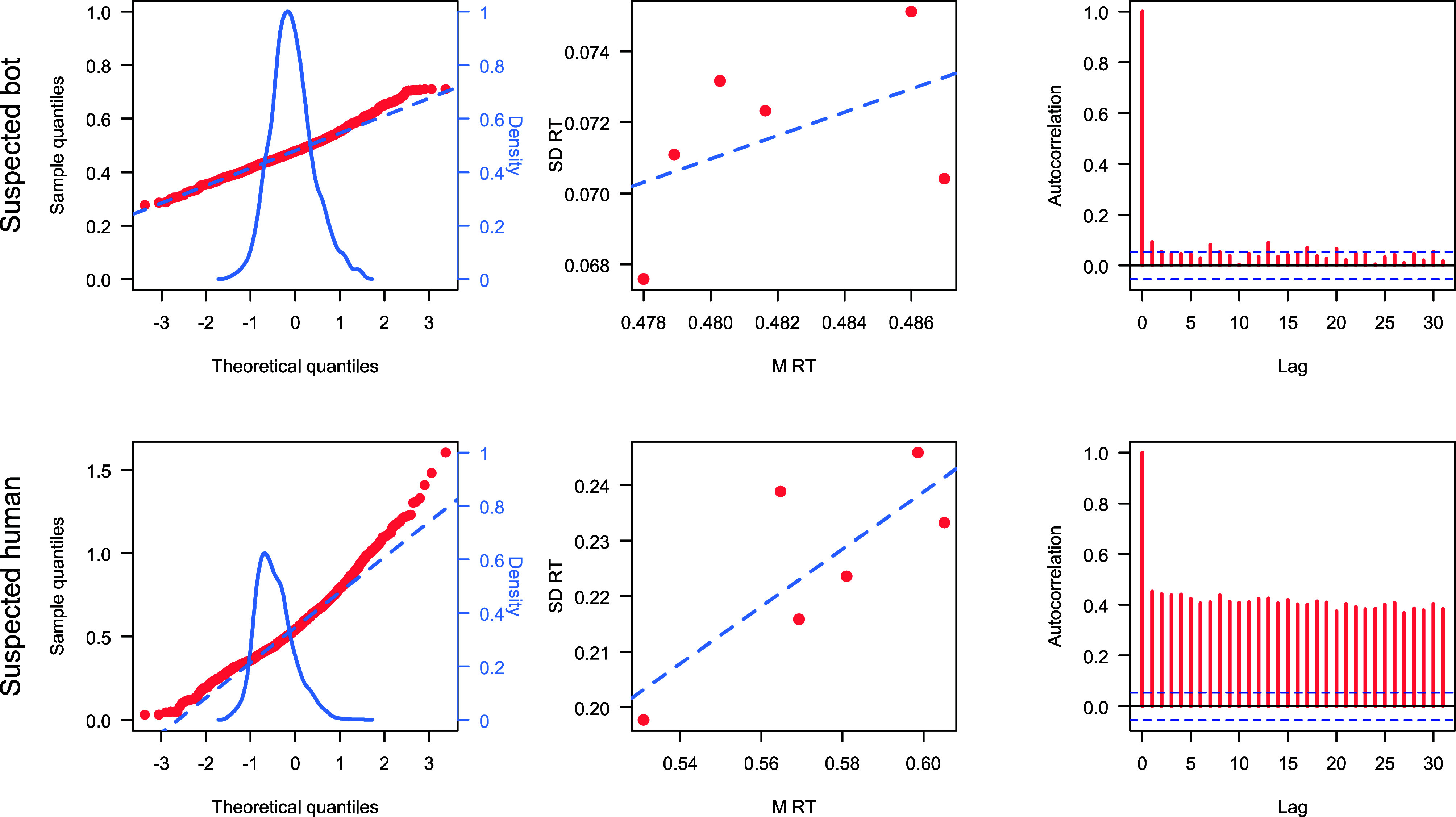
*Top*: data of a participant that may be a bot. *Bottom*: data of a most likely human participant. Left: Q–Q plot of observed RT distribution against a Gaussian distribution. Observed distribution overlaid for illustration. Middle: Correlation between mean (M) RT and SD of RT across the six experimental conditions. Note the differences in scale between the two individuals. *Right*: Autocorrelation over 30 trials. Horizontal dashed lines indicate confidence interval of an uncorrelated series.

Several participants showed behavior that was close to the simulated bot profile (Fig. 1, *Top*). Importantly, not all suspicious signatures occurred in the same individuals, highlighting that bots may vary in sophistication and the most advanced cases may already mimic some properties of human-like RT structure.

We therefore call for routine screening of all online RT datasets (including those already published) for at the very least distributional shape, mean-variance scaling, and trial-wise autocorrelation. Until such checks are standard, our field cannot confidently assume that recently reported online cognitive effects reflect human data. As models improve, RT/behavior-aware bots may begin to mimic skew, variance, and even autocorrelation. Unfortunately, we see parallels to online survey data that until recently were deemed safe, using safeguards that are now shown to fail (1, 5). When that happens, statistical sanity checks will no longer be sufficient: Our field will need methods that verify the presence of a person, not of a plausible signal generator.

We hope this expands the discussion and accelerates development of methodological safeguards, as well as editorial and citation practice of inferences based on online behavioral experiments before automated respondents silently reshape the foundations of cognitive science.
